# Treatment Preferences for Acute Allergic Reactions: A Discrete Choice Experiment

**DOI:** 10.36469/001c.117589

**Published:** 2024-06-04

**Authors:** Sofia Löfvendahl, Emelie Andersson, Sara Olofsson, Karin Wahlberg, Leif Bjermer, Göran Tornling, Jonas Hjelmgren

**Affiliations:** 1 1The Swedish Institute for Health Economics, Lund, Sweden; 2 The Swedish Institute for Health Economics (IHE), Lund, Sweden; 3 Department of Respiratory Medicine and Allergology Lund University, Lund, Sweden; 4 Respiratory Medicine Division, Department of Medicine Solna Karolinska Institutet

**Keywords:** allergies, oral dissolvable film, corticosteroids, discrete choice experiment, willingness to pay

## Abstract

**Background:** Timely treatment of acute allergic reactions (AARs) is important to minimize reaction severity. Corticosteroid tablets dissolved in water are commonly used in mainstay treatment. A new oral film that dissolves on the tongue provides a faster and less cumbersome alternative to tablets for corticosteroid administration during AARs. This study evaluated patients’ preferences for attributes related to administration mode of corticosteroids in AARs.

**Methods:** A web-based survey was sent to a sample from the adult Swedish population (≥18 years) with experience of corticosteroid treatment for AAR. We assessed the willingness to pay (WTP) for attributes related to corticosteroid treatment by applying a discrete choice experiment (DCE) approach. DCE attributes were administration mode, time to symptom relief, and price. The WTP for each attribute was derived using the attribute’s coefficient in a logistic regression analysis. We specified a forced choice (FC) and an unforced choice (UC) model. In the FC model, the respondents chose between 2 hypothetical treatments and in the UC model, between any of 2 hypothetical treatments and their current treatment.

**Results:** The final study population included 348 subjects, of which 80% were women. All the evaluated DCE attributes were significant predictors for the treatment choice (p<.001). In the FC model, the incremental WTP for an oral film compared with tablets was 409 Swedish kronor (SEK [≈€36.7]), with no other factors considered. In the UC model, the incremental WTP for the oral film compared with tablets was 574 SEK (≈€51.7). After considering the value of the respondents’ current treatment, the WTP for the oral film decreased to 336 SEK (≈€30.3). The total WTP was reduced by 17 SEK (≈€1.5) per minute of shorter time to symptom relief. Subgroup analyses showed that people with circulatory symptoms and experience of swallowing difficulties related to allergy medication had higher WTP for the oral film than the average respondent.

**Conclusion:** The findings show a substantial economic benefit of the oral film vs tablets for patients with AARs in Sweden. This result remained also after compensation for the full value of the patients’ current treatment.

## BACKGROUND

Allergic reactions can affect different organ systems and manifest as a range of symptoms. Although the reactions can last for days, they are usually more short-term and may resolve within minutes or hours^1^ but can nevertheless have a solid impact on patients’ health-related quality of life (HRQoL).^2^ Timely access to treatment is important, particularly in the case of acute allergic reactions (AAR). In AARs, symptoms arise shortly after the exposure^3^ and the severity is a continuum from mild itching of the eyes, nose, mouth, and throat to the potentially life-threatening highest grade of anaphylaxis (ie, anaphylactic shock).^4^

In general, systemic corticosteroids (betamethasone, dexamethasone, or prednisolone) in combination with antihistamines is the mainstay treatment for moderate to severe AARs not fulfilling the criteria for anaphylaxis,^5,6^ whereas only antihistamines are generally recommended for mild AARs. In contrast, the first-line treatment for anaphylaxis is intramuscular epinephrine; antihistamines and systemic corticosteroids are most often given as complements. In case of an AAR, systemic corticosteroids may be used as a rescue medicine in addition to antihistamines and epinephrine.^6–8^ Corticosteroids given for an AAR are usually administrated as tablets dissolved in water, a preparation that can be cumbersome and stressful in an acute situation. A person with an AAR may also have difficulties swallowing the medication.

Preferences for different modes of drug administration in allergy have been studied, for example, in allergic rhinitis^9^ and asthma.^10^ Both studies indicate that patients’ perspective about modes of administration are of value in the discussion about treatment schemes between the clinician and the patient.

In AAR, corticosteroids for self-administration have until now been offered only as tablets. Recently, an oral film with dexamethasone (with the same mechanism of action as conventional tablets) that dissolves on the tongue without the need for water was introduced. This mode of administration may provide benefits over conventional treatments by improving comfort and safety for patients experiencing AARs.

Patient benefit and perceived value associated with a treatment can be estimated using health economic evaluations. Usually, such analyses are based on gains in survival and health expressed in quality-adjusted life-years (QALYs).^7^ However, in the case of AARs, when patient benefit involves short-term effects or affects non-health-related aspects of patient well-being (eg, convenience, safety, and complexity of administration), QALYs are not optimal from a methodological point of view; the available health state classification systems such as EuroQol–5 dimensions (EQ-5D), are not designed to describe such acute situations and are therefore not flexible enough to provide relevant utility weights. Instead, patient preferences related to AARs should preferably be estimated by stated preference methodologies to assess the willingness-to-pay (WTP) for a medical intervention vs a comparator.^8,9^ The WTP approach can be used to evaluate the value of such events, where researchers can construct hypothetical situations that are perceived as relevant for the patient. By asking the patient what they are willing to pay for an improvement of their treatment, the utility can be expressed generically, in monetary terms, which is a clear advantage to the QALY.

The objective of this study was to evaluate patients’ preferences for AAR treatment by investigating attributes related to the administration mode of corticosteroids. Conventional treatment with tablets was compared with an oral dissolving film, which is a new administration mode of corticosteroid treatment in AARs. The WTP method, applying a discrete choice experiment (DCE) approach, was used, as the context relates to treatment decisions in short-term acute events: a situation where the traditional QALY method has proven to be less suitable.

## METHODS

### Study Design

This cross-sectional study used a 2-part web-based survey to collect data. The study population included people with self-reported allergic problems who had ever been prescribed corticosteroid tablets for AARs. The first part included background questions about patients’ characteristics, treatment, disease burden, and unmet needs. In the second part, we assessed the WTP for attributes related to corticosteroids treatment by applying a DCE approach. Before starting to answer the questionnaire, the respondents were informed about the study and gave their informed consent to participate. The focus in this article is on the WTP analysis. Results for the first part of the questionnaire have been presented in detail elsewhere.^11^

### Sample Recruitment and Selection

The study subjects were identified from a database (web-based panel) where approximately 3000 persons (Feb. 1, 2022) had stated that they have some form of allergy and therefore received information about this study. In addition, a national, study-specific advertising campaign was published on social media platforms. The recruitment and data collection started July 1 and ended mid-August 2022. All data were anonymized, and the study was approved by the Swedish Ethical Review Authority (Dnr 2022-02147-01). Informed consent was collected from all participants, and only complete responses were used in the analysis. The inclusion criteria for participating in the study were age of 18 years or older, self-reported allergic problems, and experience of an AAR that required the use or prescription of corticosteroid tablets (eg, betamethasone, dexamethasone, or prednisolone).

### DCE Experimental Design

The DCE is an established method to elicit patient preferences, and research has shown that the method provides reasonable predictions of health-related behaviors.^12,13^ The design of the DCE followed the Professional Society for Health Economics and Outcomes Research (ISPOR) good practice.^14^ Below is a description of key considerations in the context of the current study.

**Attributes**: The focus was the choice between different hypothetical treatments for AARs based on real-life treatment–specific attributes such as administration mode, time to symptom relief, and the price the patient is willing to pay for the medicine. A pragmatic approach was applied for the selection of attribute and the number of levels within each attribute. Only attributes of relevance for the choice context were included, and attributes such as adverse events and risk of an allergic reaction were omitted. Adding a side effect attribute would probably provide limited value since both treatments contain the same active substance class and thus affect patients similarly. Furthermore, we assumed that the respondent is aware of his/her own risk of an allergic reaction. The relevance of included attributes and levels were validated by a clinical expert, and the survey was tested in a pilot version (both by people with and without experience of AARs) prior to finalization.

For the *administration mode attribute*, the respondents were asked to choose between “tablets to be dissolved in water” and an oral dissolving film (**Table 1**). This attribute was intended to weigh in aspects such as convenience, safety, and accessibility of the alternatives. The respondents made a choice between alternatives based on their own experience of treatment with tablets and the description of the oral dissolving film provided in the questionnaire.

The *time to symptom relief* was expressed as “how many minutes it will take before the patient feels symptom relief” and included the time it takes to prepare and swallow the medicine. In the pilot, we involved patients and clinical experts to establish a relevant time interval and ended up with 5 levels ranging from 10 minutes to >45 minutes (**Table 1**).

**Table 1.** Attributes and Levels Used to Construct Alternative Treatment Options

**Attribute**	**Level**
Administration modes	Tablets dissolved in water
	Oral dissolving film
Price per acute attack (payment by the patient), SEK	1000
	500
	250
	100
	45
Time to symptom relief (min)	10
	15
	20
	30
	45

Note: 100 SEK ≈€9.

The *price attribute* should consider a wide range of price levels to capture individuals with a low or high WTP for symptom relief a new administration form, respectively. The price attribute in the present experiment included 5 levels ranging from 45 to 1500 SEK (Swedish kronor) [≈€4 to €135] and illustrate a hypothetical price that the patient should pay (**Table 1**). The price level of 45 SEK is slightly above the market price for 6 or 7 tablets of dexamethasone. The price of 1500 SEK is considerably higher than the current market prices and was included to test the highest WTP for options with a favorable profile in terms of time to symptom relief and mode of administration.

**Choice-set experiment design:** The design, in all, enabled 50 different choice combinations (2 × 5 × 5 = 50). However, it would be practically challenging to have a questionnaire with 50 choice questions. Therefore, to obtain an orthogonal design (to minimize multicollinearity) with fewer combinations, we used an orthogonal catalog system developed by Hahn et al,^15^ which generated a design with 16 combinations (16 choices). Effect coding was based on the approach suggested by Hensher et al.^16^

Thereafter, the choice sets were randomly paired to create a tradeoff between choice sets. This process was conducted until there were no duplets (no tradeoff between alternatives) or dominance between choice alternatives (ie, one choice set more favorable in all attributes). To minimize the probability of cognitive overload, the 16 choice alternatives were divided into 2 blocks with 8 choice alternatives in each block and 2 versions of the questionnaire.

**Figure 1.**
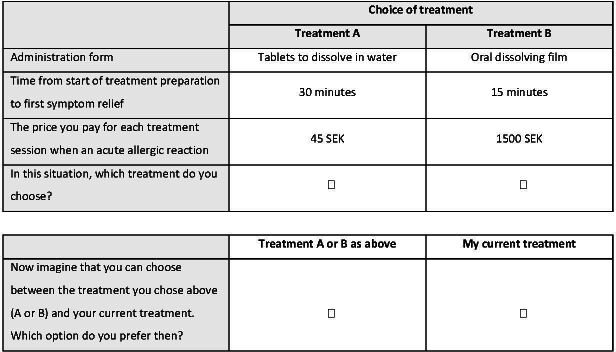
Example of Choice Situation with Forced Choice Alternatives and “Status Quo” Choice Alternative Note: 100 SEK≈€9.

The last step in the design was to establish the choice situations. In DCE analyses regarding medical services, evidence indicates a relatively high preference for services that respondents have experienced, a “status quo” (SQ) state.^17^ To ensure that choice situations are perceived as realistic and capture a real-life choice behavior, we decided to include an SQ alternative where the respondent could choose not to make a choice between “A or B” (forced choice) but instead choose their current treatment (unforced choice). This SQ alternative can be referred to as the status quo and is the participant’s reference point or current situation^18^ **(Figure 1**). This SQ alternative should be represented by attribute levels that most closely describe the respondents’ current treatment, provided that the SQ can vary for respondents.^19,20^

**“Protest” respondents:** A “protester” is a respondent who does not make the required trade-offs when making their choices, and the expressed choices do not genuinely reflect their true preferences and values.^21^ Protest respondents in our study were classified as (1) those who prefer A or B but at the same time expressed that the public sector should bear all costs, (2) those who randomly chose different options, and (3) those who for all 8 situations chose the same alternative (only A or only B). All respondents classified as protesters were excluded from the analyses.

**Data analysis:** The DCE questions generated panel data (everyone responded to 8 choice sets). The coefficients of the included attributes in the DCE experiments were analyzed using conditional logistic regression with random effects (command ‘xtlogit’ in STATA). The models assumed linearity and independence of observations, which may not hold true in all cases and could affect the accuracy of the estimated coefficients and WTP values derived from the analysis. We specified a forced choice (FC) model (**Equation 1**) and an unforced choice (UC) model (**Equation 2**).

(1)Y=α+βxadminmode+βytime+βzprice+ε

(2)Y=α+βxadminmode+βytime+βzprice+βsASC+ε

The dependent variable Y was defined as choosing a hypothetical treatment profile (0 = not choosing a treatment profile, 1 = choosing a treatment profile) and ε was the error random term. The independent variables (and coefficients $\beta_{x}$, $\beta_{y}$ and $\beta_{z}$) were characteristics of the hypothetical treatment alternatives (ie, administration form [0=tablets; 1=oral dissolving film]), time to symptom relief (10, 15, 20, 30, and 45 minutes) and price of treatment (45, 100, 250, 500, and 1500 SEK) (**Equation 1**). In the UC analysis, the respondents’ own treatment was considered by inclusion of the alternative specific SQ constant (coefficient $\beta_{s}$) (**Equation 2**), representing the respondents’ current treatment (0 = treatment A or B, 1 = own treatment) (**Figure 1**).

To find the respondents’ own SQ values, the questionnaire asked the respondents to state the time span for preparation and swallowing of allergy medicine and time span from swallowing until first relief of symptoms, respectively. These time span questions related to the respondents’ last acute allergic reaction and appeared in the questionnaire prior to the choice sets not to bias the attribute levels for the SQ state. The individual mean number of minutes for the entire time span was used as the time attribute level for the SQ state. The price attribute level in the SQ alternative was set to 0. In the model, the SQ variable was coded as a dummy variable (0 = treatment A or B, 1 = my current treatment). In the questionnaire, we did not ask about the respondents’ current treatment; hence “current treatment” must be seen as a variety of unknown treatments.

The DCE estimates the marginal rate of substitution (MRS) between the different attributes and their levels. MRS is the relationship where an individual choice to make a trade-off between 2 goods. The MRS is calculated as $MRS_{ij}=X_{i}/X_{j}$ where i and j are 2 different goods. By including a monetary attribute, it is possible to derive the WTP for the nonmonetary

attributes. This is done by scaling the coefficient of interest with the price coefficient and multiplying by -1 (**Equation 3**) where $\beta_{x}$ is the coefficient of the attribute of interest and $\beta_{price}$ is the price coefficient.

(3)WTPx=−βxβprice

To further investigate how preferences varied across respondent groups, we conducted a subgroup analysis of the UC model. Of particular interest was the evaluation of an oral dissolving film compared with tablets, and therefore we included an interaction term between the attribute “administration mode” and different subgroups in the regression. We performed 2 subgroup regression analyses:

1. A full model including all background variables (**Online Supplementary Material**)
2. A reduced model including statistically significant variables (*p*<.05) from the full model analysis.

The reduced model is presented in the main text and the full model in the **Online Supplementary Material**. A *p* value <.005 was considered statistically significant.

## RESULTS

### Demographic and Socioeconomic Characteristics

The survey was sent to 830 subjects and completed by 426 eligible respondents. Of these, 39 (9%) who stated that they had allergy only to pollen were excluded from analyses. This exclusion assumed that respondents with allergy to pollen mainly use corticosteroids for preventive treatment of allergic symptoms (as opposed to acute treatment in moderate to severe AARs).

For the current analysis, 39 subjects (9%) defined as “protesters” were also excluded (**Online Supplementary Material**). Hence, the final study sample of eligible respondents was 348. Mean (SD) age of the study sample was 41 (13.8) years; 80% were women (**Table 2**). Fifty percent had a university education, and 70% were working full-time or part-time. Household income varied, and just under half of the subjects stated a monthly income below 40 000 SEK (≈€3590).

### Allergic Reaction and Treatment Situation

Half of the respondents (51%) had experienced their most recent AAR less than a year ago (**Table 2**), and the mean (SD) perceived degree of severity on a scale from 0 (very mild) to 10 (very severe) was 7.1 (2.0). Moreover, at the most recent AAR, the majority (66%) had taken fewer than 10 corticosteroid tablets, with a median (Q25; Q75) time required to prepare and swallow the allergy medicine of 2.5 (0.5; 3.1) minutes. The time from swallowing the medicine until first relief of symptoms was 17.5 minutes (12.5; 25). A quarter (25%) of the respondents experienced difficulties swallowing their allergy medicine when having an AAR. Two hundred twenty-six (65%) respondents had experienced not having the allergy medicine immediately available when needed for treatment of an AAR. Of those, nearly 80% had not retrieved the medicine from the pharmacy and 20% had been away from home without bringing the medicine. On a scale from 0 (not worried at all) to 10 (very worried), the mean (SD) perceived degree of worries about having an AAR was 4.3 (2.6).

**Table 2.** Patient Characteristics (n = 348)

**Characteristics**	**n (%)**
Sex	
Female	277 (79.59)
Male	71 (20.40)
Age (y)	
Mean (SD)	40.86 (13.79)
Median (Q25, Q75)	39 (29; 51.5)
Level of education (%)	
Compulsory school	10 (2.87)
Upper secondary school	94 (27.01)
University <3 y	54 (15.52)
University >3 y	173 (49.72)
Other	17 (4.89)
Work force participation (%)	
Working (full-time or part-time)	243 (69.83)
Other (retired, student, on sick leave)	105 (30.17)
No answer	
Household income (SEK monthly before tax) (%)	
≤9999	12 (3.45)
10 000-19 999	43 (12.36)
20 000-29 999	48 (13.79)
30 000-39 999	65 (18.68)
40 000-49 999	35 (10.06)
50 000-59 999	30 (8.62)
60 000-69 999	29 (8.33)
70 000-79 999	22 (6.32)
≥80 000	44 (12.64)
Prefer not to say	20 (5.75)
**When was the last time you had a severe allergic reaction? n (%)**	
<6 months ago	87 (25.00)
Between 6 months and 1 year ago	90 (25.86)
>1 year ago	166 (47.70)
Do not know	5 (1.44)
**How severe was your last acute allergic reaction? (0-10 scale, where 0=very mild, 10=very powerful)**	
Mean (SD)	7.13 (1.96)
Median (Q25, Q75)	7 (6; 8)
**How many corticosteroid tablets did you take the last time you had an acute allergic reaction? n (%)**	
<5	109 (31.32)
5-10	122 (35.06)
11-15	88 (25.29)
16-20	24 (6.90)
>20	5 (1.44)
Median (Q25, Q75) (cortisone tablets)	7.5 (2; 13)
**Approximately how long did it take to prepare and swallow your allergy medication at last time you had an acute allergic reaction? n (%)**	
<1 min	92 (26.44)
1-4 min	157 (45.11)
5-10 min	61 (17.53)
>10 min	10 (2.87)
Do not know	28 (8.05)
Median (Q25, Q75) (min)	2.5 (0.5; 3.1)
**How long does it normally take from the time you swallow your allergy medication to start feeling relief of symptoms?**	
<10 min	38 (10.92)
10-15 min	81 (23.28)
15-20 min	80 (22.99)
20-30 min	62 (17.82)
30-45 min	27 (7.76)
>45 min	17 (4.89)
Do not know	43 (12.36)
Median (Q25, Q75) (min)	17.5 (12.5; 25)
**No. of acute allergic attacks treated with corticosteroids last year**	
Mean (SD)	1.64 (2.01)
Median (Q25, Q75)	1 (0; 2)
**Experience of difficulty swallowing allergy medicine when having an acute allergic reaction? (%)**	
Yes	89 (25.57)
No	244 (70.11)
Do not know	15 (4.31)
**Experience of not having the allergic medicine immediately available when need for an acute allergic reaction**	
Yes	226 (64.94)
No	110 (31.61)
Do not know	12 (3.45)
**Worry about having an acute allergic reaction (0-10 scale: 0 = not worried at all, 10 = very worried)**	
Mean (SD)	4.28 (2.56)
Median (Q25, Q75)	4 (2; 6)

Note: 100 SEK ≈ €9.

Abbreviations: Q25, lower quartile; Q75, upper quartile.

### Preferences and WTP for the Whole Population in the FC Model

**Table 3** shows the results of the FC model. The estimated regression coefficients for administration mode, time from start of treatment preparation to first symptom relief (per minute), and price had a statistically significant impact on the choice of treatment in the event of an AAR. The positive regression coefficient for administration mode means that, all else being equal, the probability of choosing a given treatment in the event of an AAR increases if one can choose an oral dissolving film as the administration mode. The negative regression coefficient for time means that the probability of choosing a given treatment in the event of an AAR decreases for each extra minute from start of medication preparation to first symptom relief.

**Table 3.** Random Effects Logit Regression Results and Estimated Willingness to Pay (n = 348): Forced Model

**Attributes**	**Forced Model**				
	**β**	***P* Value**	**95% CI**		**WTP (SEK)^a^**
Administration mode (tablets = 0)	0.995336	<.001	0.8849225	1.105749	409
Time (per min)	-0.0554436	<.001	-0.0632872	-0.0476	-23
Price (SEK/dose)	-0.0024349	<.001	-0.0026418	-0.0022214	--
Constant	1.942005	<.001	1.68445	2.19956	798
Observations	5568				
Groups of observations	2784				
Log likelihood function, *L*	-3169				
Restricted log likelihood function, *L_0_*	-3859				
McFadden’s R^2^ (1−*L/L_0_*)	0.18				

^a^The marginal rate of substitution between price per dose and characteristic $i=\beta_{i}/-\beta_{price}$ (=WTP), where $\beta_{i}$ denotes the regression coefficient for the characteristic i and $\beta_{price}$ denotes the regression coefficient for price.

Note: 100 SEK ≈€9.

Groups of observations=total number of choice situations for 348 respondents.

Abbreviations: SEK, Swedish kronor; WTP, willingness to pay.

The WTP of 409 SEK [≈€36.7] for an oral dissolving film should be interpreted as the additional value the average respondent is willing to pay to get that mode of administration compared with tablets when having an AAR. The WTP of 23 SEK (≈€2.1) for time should be interpreted as the amount of money the average respondent needs as compensation for each additional minute it takes to prepare and swallow the drug.

### Preferences and WTP for the Whole Population in the UC Model

**Table 4** shows the results of the UC model. As was the case with the FC model, all included variables had a statistically significant impact on the choice of treatment in the event of an AAR. The significance of the SQ-state coefficient indicates that the respondents are not indifferent between staying with their current treatment and changing to 1 of the 2 hypothetical alternatives when the attributes are accounted for.

**Table 4.** Random Effects Logit Regression Results and Estimated Willingness to Pay (n= 348) Respondents: Unforced Model

**Attributes**	**Unforced Model**				
	**β**	***P* Value**	**95% CI**		**WTP (SEK)^a^**
Administration mode (tablets = 0)	1.100989	<.0001	0.9848094	1.217169	574
My own treatment (treatment A or B = 0)	0.4572243	<.0001	0.3482703	0.5661783	238
Time (per min)	-0.0328119	<.0001	-0.0355715	-0.0300523	-17
Price (SEK/dose)	-0.0019216	<.0001	-0.0020904	-0.0017529	--
Observations	8,352				
Groups of observations	2,784				
Log likelihood function, *L*	-4745				
Restricted log likelihood function, *L_0_*	-5316				
McFadden’s R^2^ (1−*L/L_0_*)	0.11				

^a^The marginal rate of substitution between price per dose and characteristic $i=\beta_{i}/-\beta_{price}$ (=WTP), where $\beta_{i}$ denotes the regression coefficient for the characteristic i and $\beta_{price}$ denotes the regression coefficient for price.

Note: 100 SEK ≈ €9.

Groups of observations = Total number of choice situations for 348 respondents.

Abbreviations: SEK, Swedish kronor; WTP, willingness to pay.

When comparing the coefficients of the FC and UC models, it can be noticed that the coefficients are somewhat different, which will have an effect of the trade-offs (WTP) between the choice attributes. In the UC, the WTP for the oral dissolving film was 574 SEK (≈€51.7). There was a positive WTP value for the SQ state (238 SEK [≈€21.3]), indicating a preference for current treatment. After considering that some respondents prefer to remain on their current treatment, the WTP for the oral film decreased to 336 SEK (≈€30.3). The price for each extra minute of symptom relief was 17 SEK (≈€1.5).

### Preferences and WTP in Subgroups

The above WTP analyses did not consider how different subgroups in the study population value the different attributes. As observed in **Table 2**, the study sample included subjects with different characteristics, including how they experienced their allergy problems (eg, type of allergy, symptoms, perceived anxiety, etc). Hence, we also considered how different subgroups based on patient characteristics valued the different attributes. **Table 5** shows the statistically significant results of the conditional logistic regression results (reduced model) of the subgroup analysis and estimated WTP. Complete results from both the full model and the reduced model are presented in the **Online Supplementary Material**.

**Table 5.** Random Effects Logit Regression Results of the Subgroup Analysis (Reduced Model) and Estimated Willingness to Pay

	**Reduced Model**		
	**β**	***P* Value**	**WTP (SEK)^a^**
Oral film (tablets = 0)	1.204	<.001	608
My own treatment (treatment A or B=0)	0.424	<.001	214
Time (min)	-0.035	<.001	-17
Price (in SEK)	-0.002	<.001	NA
**Interaction variable between variables below and administration mode = 1 (0 = tablets, 1 = oral dissolving film)**			
Sex (male=0, female=1)	-0.453	.003	-229
Age	0.009	.027	5
Circulatory symptoms (no = 0, yes = 1)	-0.340	.014	-171
No. of AAR treated with cortisone during the last year	-0.097	.002	-49
Difficulties swallowing allergy medicine during AAR (no = 0, yes = 1)	0.502	.000	253
AIC	9044		
Log likelihood function, *L*	-4507		
Restricted log likelihood function, *L_0_*	-5316		
McFadden’s R^2^ (1−*L/L_0_*)	0.152		
Observations	7992		
Groups of observations	2664		
**No. of individuals**	**333**		

Note: 100 SEK ≈€9.

Groups of observations= total number of choice situations for 333 respondents.

Abbreviations: AAR, acute allergic reaction; AIC, Akaikie information criterion; SEK, Swedish kronor; WTP, willingness to pay.

The subgroup analyses show that female respondents, compared with men, had a lower probability of choosing an oral dissolving film as administration mode, with a WTP of -229 SEK (≈€20.5). A corresponding negative association was also observed for respondents with circulatory symptoms (vs no circulatory symptoms) and with increasing numbers of AARs treated with corticosteroids during the last year. Respondents with swallowing difficulties had a higher probability of choosing an oral dissolving film as administration mode in the event of an AAR than those reporting no such difficulties, with an WTP of +253 SEK (≈€22.7).

## DISCUSSION

The main purpose of this study was to assess the economic value of a novel oral dissolvable film compared with tablets for the treatment of AARs in adults prescribed corticosteroids in Sweden. Patients who are prescribed corticosteroids due to allergic problems are mainly those with moderate to severe disease activity. However, in Sweden, the size of this patient group is unclear. It has been estimated that more than 30% of the population suffer from some type of allergy in Sweden.^22^ According to the Swedish National Prescribed Drug Register, 31 894 patients per year were prescribed an adrenaline pen in 2018-2021.^23^ In our study, all subjects had been prescribed corticosteroids for AARs, and 31% also had a prescription for adrenaline. If these figures are applied to Sweden as a whole, the allergy population with moderate to severe disease activity would include approximately 103 000 individuals, constituting 3% to 6% of the total Swedish population with allergy.

One main study finding was that the oral dissolvable film was perceived as more attractive than tablets (*p*<.001) with a higher monetary value in both the FC and the UC model. However, the monetary value (per acute reaction) of the oral film vs the tablets was greater in the UC model (SEK 574 [≈€51.5]) compared with the FC model (SEK 409 [≈€36.7]). Previous research has demonstrated that inclusion of an SQ alternative (ie, current treatment) adds to the realism of choice situation and has an impact on the monetary value of the included alternatives. An SQ alternative should therefore be included to provide a real-life baseline.^19^ Of 348 respondents, 282 (81%) opted for “my current treatment” in at least 1 of the choice situations, which means that some of the alternatives presented to the respondents were regarded as inferior compared with respondents’ current treatment. We noted that respondents tended to choose their current treatment more frequently when both alternatives, A and B, comprised tablets (58%) than when at least 1 of the alternatives comprised the oral film (38%). This is one important explanation of why the value of the oral film was greater (574 SEK vs 409 SEK [≈€51.5 vs ≈€36.7]) when respondents could choose an SQ alternative if an inferior alternative was presented to them. After considering that some respondents prefer to stay with their current treatment in the UC model, the WTP for the oral film decreased to 336 SEK (≈€30.3).

Theoretically, the UC model could enable comparative analysis of current treatment vs the oral film that would be acceptable from a health technology assessment (HTA) perspective. In our study, the monetary value of current treatment was estimated to SEK 238 [≈€21.3]. It is, however, not known what current treatment represents in our study. All respondents had been treated with corticosteroids for an AAR. Further, 31% has been prescribed an adrenaline pen, which is an important component for the treatment of an acute and severe allergic reaction with risk for anaphylaxis. In addition, it is not known to what extent the respondent uses their corticosteroid tablets and other allergy treatments (eg, antihistamines, alternative medicines). To qualify for a HTA, a more systematic recording of the respondents’ current treatment would have been required to assess the value of this alternative vs the oral film.

Earlier DCE studies have shown a positive relationship between the risk of disease activity/illness and the WTP, meaning that individuals are willing to pay more if the risk is high.^24^ At the same time, there is a negative relationship between side effects and the WTP, implying that individuals to some extent will be compensated for treatment to take the medication.^25^ This could help to understand nonadherence behavior in a patient population. Our study did not include any of these attributes. As we only included patients with allergic reactions who were assumed to be familiar with their own disease activity and symptoms, we assumed that they were also familiar with their risk profile. If individuals with a high risk of having an AAR view the oral film as a rescue medication, they would be willing to pay more for the oral film than those with a lower risk. However, the results in our study provide ambiguous support to this hypothesis. For instance, respondents who were highly insecure with their current treatment were not willing to pay more for the oral film, nor were patients who were highly worried of having an AAR (*p*>.05). Also, respondents who had experienced more AARs during last year had slightly lower WTP (*p*<.05). One interpretation of these results is that individuals do not see large differences between the treatments with regard to symptom relief, as the oral film and the tablets contain the same active substance class.

On the contrary, higher WTPs for the oral film were observed for respondents with swallowing difficulties (*p*<.0001), whereas women had a lower WTP (*p*<.0001). As the purpose of the oral film is to facilitate for patients to swallow the medication, it is not surprising that individuals with swallowing difficulties would be willing to pay more. A priori, it is not obvious that women would be willing to pay less than men; this result is surprising considering that women chose the oral film to a greater extent than men (75% vs 68%) if the price of the oral film and tablets was the same. Women’s lower WTP compared with men implies that women reacted more to the price attribute in the DCE than men did.

As the study sample was recruited from a web panel and social media and hence not population-based, generalization outside the study sample is limited, and there might be a self-selection bias. This may be of particular importance regarding the sex distribution in our study sample, which included mostly females (80%). This is probably a higher percentage than in the target population, suggesting that the average WTP would be somewhat higher in the target population, which presumably has a higher proportion of men.

The study survey was sent to 830 individuals and completed by 426 eligible respondents. Of these, 39, who stated they had allergy only to pollen, were excluded from the analysis. In addition, 39 individuals who was classified as protesters were excluded, leaving a total sample of 348 individuals. The rationale for excluding individuals with only pollen allergy was that they are more likely to use a combination of corticosteroids and antihistamines to prevent allergic reactions. In terms of protesters, earlier DCE studies have discussed to what extent they may impact the overall study results.^19,26,27^ In line with earlier recommendations for conducting DCE studies, we excluded protesters from our main analysis.^21^ The removal of protesters should not have biased the results, as the protesters were similar to the overall sample regarding key variables such as sex, age, education, and work participation. However, the proportion of responders with more than 3 concurrent allergies (eg, food, fur, and insects) were lower among the protesters compared with the total sample (28% vs 43%). In this study, due to relatively small sample sizes of the excluded groups, the result of including these groups was impacted to a minor extent; the WTP for the oral film decreased from 574 to 540 SEK (≈51.5 to ≈€48.4) if both groups were included, to 563 SEK (≈€50.5) if only pollen allergy was excluded and to 550 SEK (≈€49.3) if only protesters were excluded. All analyses were conducted using the same UC logit regression model as was used in the main analysis.

Another aspect regarding how the inclusion of different groups affects the results relates to age. In this study, only individuals over the age of 18 participated. There is reason to believe that inclusion of children may have increased the WTP for the oral film, as there is some evidence in the literature that indicates higher WTP within populations of children compared with adult populations.^28^ This is an aspect that should be addressed in further research. It has been argued that the stated preference methodology, such as DCE, is especially suitable for assessment of acute conditions, where it is difficult to design clinical trials with the purpose to capture the health impairment of a short-term acute event.^29^ Usually, the EQ-5D is used in clinical trials to provide healthcare profiles that are translated to health-related quality of life (HRQoL). Healthcare profiles are often quite stable over time and are not designed to illustrate instant changes in HRQoL, which may occur infrequently and be of very short duration (eg, acute events). To illustrate, let us assume that a subject with allergy in “full health” gets a moderate allergic reaction that results in a health state that can be translated to the EQ-5D health profile ‘12222’ (QALY weight 0.620)^30^ and the duration of the attack after treatment is 6 hours. The annual utility loss for 1, 2, and 3 such attacks would be 0.00026, 0.00052, and 0.00078, respectively. Hence, it may require a very large data set of patients over a long time to register enough events to be able to detect differences in HRQoL across patients in the treatment arms. Another challenge is that the gains in utility would be very small, and it is therefore questionable whether HTA organizations would accept the evidence.

In our study, respondents experienced mean 1.6 attacks per year, and each attack was on average moderate to severe (mean 7.1 on a 1-10 scale). The annual gain in HRQoL of using the oral film vs tablet would probably, based the argument above, be very small. Still, these responders would be willing to pay a considerable amount, 409 SEK (≈€45.4) per acute attack, for the oral film. When the respondents were asked why they preferred the oral film over the tablets, 25% answered that it was easy to “bring the medicine with them” and 51% answered that the medicine was “easy to take.” These answers from potential users confirm evidence from earlier research that aspects such as “insurance value” (“safety”) and “process utility” (delivery of care) are important attributes from an individual utility perspective.^31–34^ These attributes are not captured by the traditional QALY approach.

In conclusion, the findings show a substantial economic benefit of the oral film vs tablets for patients with AARs in Sweden. This result remained also after compensation for the full value of the patients’ current treatment. The awareness of patients’ preferences for different treatment choices for AARs may improve both treatment adherence and patient outcomes.

### Disclosure

G.T. is a member of the board of AcuCort AB.

## Supplementary Material

Online Supplementary Material
