# Supplementary material for: Treatment Preferences for Acute Allergic Reactions: A Discrete Choice Experiment
Source: J Health Econ Outcomes Res. 2024 Jun 4;11(1):157–65. doi: 10.36469/001c.117589 (PMC11156467; doi:10.36469/001c.117589)
Supplement: Online Supplementary Material [file jheor_2024_11_1_117589_230443.pdf]

Journal of Health Economics and Outcomes Research

Online Supplementary Material

Treatment Preferences for Acute Allergic Reactions: A Discrete Choice Experiment. *JHEOR*. 2024;11(1):157-165. doi:10.36469/jheor.2024.117589

Table S1: Exclusion of Subjects from the Final Study Sample

Table S2: Random Effects Logit Regression Results of the Subgroup Analysis (Reduced Model) and Estimated Willingness to Pay

This supplementary material has been provided by the authors to give readers additional information about their work.

This is an open-access article distributed under the terms of the Creative Commons Attribution 4.0 International License (CCBY-4.0). View this license's legal deed at http://creativecommons.org/licenses/by/4.0 and legal code at http://creativecommons.org/licenses/by/4.0/legalcode for more information.

Of the 426 subjects in the initial study sample, 78 (18.3%) were excluded (Table S1).

Table S1. Exclusion of Subjects from the Final Study Sample

	N	Comment
Only pollen allergy		
Yes	39	Excluded
Why did you choose your own treatment in all scenarios? (of those who chose "My current treatment in all choice sets")		
I value another treatment, but I think the public should bear the cost	8	Excluded as protesters
What did you consider when choosing between different options? (of those who did not chose "my current treatment in all choice sets")		
I answered randomly	2	Excluded as protesters
Individuals who answered the same treatment option (A or B) for all 8 choice situations		
Alternative A	29	Excluded as protesters

Preferences and willingness-to-pay in subgroups (Table S2)

Table S2. Random Effects Logit Regression Results of the Subgroup Analysis (Reduced Model) and Estimated Willingness to Pay

	Full Model			Reduced Model		
	β	*P* Value	WTP	β	*P* Value	WTP
Oral film (tablets = 0)	2.459	.036	1193.6	1.204	<.001	607.5
My own treatment (treatment A or treatment B = 0)	0.376	<.001	182.3	0.424	<.001	213.8
Time (minutes)	-0.040	<.001	-19.4	-0.035	<.001	-17.4
Price (SEK)	-0.002	<.001	NA	-0.002	<.001	NA
Interaction variable between variables below and administration mode=1 (0=tablets, 1= mouth-dissolving film)						
Sex (male = 0)	-0.625	.001	-303.6	-0.453	.003	-228.7
Age	0.014	.014	6.8	0.009	.027	4.7
Upper secondary school (compulsory school = 0)	-0.263	.614				
University <3 years (compulsory school = 0)	-0.194	.712				
University ≥3 years (compulsory school = 0)	-0.398	.531				
Occupation (not working=0)	0.055	.765				
Log income	-0.168	.178				
Pollen allergy (no = 0)	0.115	.562				
Food allergy (no = 0)	0.107	.536				
Fur allergy (no = 0)	-0.068	.693				
Insect sting (no = 0)	-0.068	.714				
Other allergies (no = 0)	-0.222	.209				
General symptom (no = 0)	0.148	.350				
Skin symptoms (no = 0)	-0.155	.411				
Respiratory symptoms (no = 0)	0.360	.087				
Abdominal symptoms (no = 0)	0.011	.951				
Circulatory symptoms (no = 0)	-0.466	.010	-226.2	-0.340	.014	-171.4
Other symptoms (no = 0)	-0.533	.126				
Duration of allergy problems 2-5 years (<2 years = 0)	-0.198	.717				
Duration of allergy problems >5 years (<2 years = 0)	0.253	.626				
No. of AAR treated with cortisone during the last year	-0.111	.004	-53.8	-0.097	.002	-48.8
Ever prescription of adrenaline pen (no = 0)	-0.047	.793				
Difficulties swallowing allergy medicine when AAR (no = 0)	0.693	.000	336.2	0.502	.000	253.2

Table S2. Random Effects Logit Regression Results of the Subgroup Analysis (Reduced Model) and Estimated Willingness to Pay

	Full Model			Reduced Model		
	**β**	***P* Value**	**WTP**	**β**	***P* Value**	**WTP**
Difficulties swallowing in general (no = 0)	-0.482	.069				
Highly insecure about my current allergy treatment (less insecure = 0)	0.045	.821				
High worries about having an AAR (less worries = 0)	0.231	.141				
Experience of an AAR that led you to interruption of daily activities (no = 0)	0.265	.261				
Experience of an AAR that left you fearing for life (no = 0)	0.129	.492				
Experience of not having allergy medicine when needed for an AAR (no = 0)	0.181	.305				
Regular allergy healthcare check-ups (no = 0)	-0.279	.096				
Ever emergency care due to an AAR (no = 0)	-0.188	.332				
AIC	7183			9044		
Log likelihood function, L	-3524			-4507		
Restricted log likelihood function, L_0	-5316			-5316		
McFadden's R^2 ($1-L/L_0$)	0.337			0.152		
Observations	6336			7992		
Groups of observations	2112			2664		
**No. of individuals**	**264**			**333**		

Groups of observations = Total number of choice situations for the respondents.
Abbreviations: AAR, acute allergic reaction; AIC, Akaike information criterion.
